# Comparative evaluation of the laterally closed tunnel technique with connective tissue graft and collagen matrix for treating localized type 1 gingival recession: a randomized, controlled clinical trial

**DOI:** 10.1186/s12903-025-07515-9

**Published:** 2026-01-06

**Authors:** Ahmed ElBana, Wafaa Saleh, Jilan Youssef

**Affiliations:** Oral Medicine, Periodontology, Diagnosis, and Oral Radiology Department, Faculty of Dentistry, Mansoura University, Mansoura, 33516 Egypt

**Keywords:** Laterally closed tunnel technique, Connective tissue graft, Collagen matrix, Localized recession defects

## Abstract

**Objective:**

There is limited evidence about the short- and long-term outcomes of the laterally closed tunnel (LCT) technique for gingival recession (GR) treatment. This clinical trial aimed to evaluate the short-term clinical effectiveness of and patient-reported outcomes of the LCT technique when used in combination with either connective tissue graft (CTG) or collagen matrix (CM).

**Materials and methods:**

A total of forty patients were randomly assigned to either the control group (LCT + CTG) or the test group (LCT + CM) for localized GR type 1 treatment. Primary outcome measures included recession depth (RD), recession width (RW), root coverage percentages (MRC%), and complete root coverage percentage (CRC%). Secondary outcomes included gingival thickness (GT), keratinized tissue width (KTW), clinical attachment level (CAL), attached gingiva width (AGW), root coverage esthetic score (RES), and patient-reported outcome measures (PROMs). Clinical evaluations were performed at baseline, 3 months, and 6 months after treatment.

**Results:**

At both 3 and 6 months postoperatively, the primary outcomes (RD, RW, MRC%, and CRC%) showed significant improvement (MRC% 93.98 ± 8.58% and 87.24 ± 13.70%, and CRC% 90% after 6 months; *p* < 0.001). All the secondary outcomes, including (GT, KTW, CAL, AGW, RES, and PROMs were also significantly improved at the end of the study in both groups (*p* < 0.001).

**Conclusion:**

The LCT technique proved to be an effective technique for localized recession type 1 treatment when used in combination with CTG or CM.

**Clinical relevance:**

Both grafting materials used in combination with the LCT technique resulted in favorable short-term clinical and esthetic outcomes for localized gingival recession.

**Trial registration:**

The current clinical trial was retrospectively registered in ClinicalTrials.gov (ID: NCT06065774) and initially released in 13-09-2023 and finally released in 18-11-2024. https://register.clinicaltrials.gov/prs/beta/studies/S000CV1L00000057/recordSummary.

**Supplementary Information:**

The online version contains supplementary material available at 10.1186/s12903-025-07515-9.

## Introduction

Gingival recession (GR) is the displacement of the gingival margin apically beyond the cementoenamel junction (CEJ) level, leading to the exposure of the root surface. Loss of gingival tissues can negatively impact the patient’s esthetics and periodontal health [1]. The clinical relevance of GR lies in root exposure and its associated hypersensitivity, root caries, and patient appearance, especially in the esthetic zone [2, 3].

Many classification systems are used to describe GR defects. According to Cairo’s classification, recession type 1 (RT1) defects are characterized by intact interproximal bone and papilla without attachment loss, where complete root coverage is predictable [4]. Various surgical approaches and biomaterials have been proposed to restore both the function and esthetics for GR management [5–10].

Among these techniques, the coronally advanced flap (CAF) remains the most used technique, providing reliable root coverage through coronally positioning the gingival margin. Its predictability increases when combined with a connective tissue graft (CTG), which improves the gingival phenotype and supports early wound healing [11–13]. However, CAF has some limitations, including esthetic distortion in deep uneven multiple recession, limited effectiveness when interproximal attachment loss is present, and the need for a second surgical site for CTG harvesting, resulting in postoperative morbidity and patient discomfort [14, 15].

To address these shortcomings, tunneling techniques have been developed as a minimally invasive, biologically oriented alternatives that preserve the interdental papillae and maintain the vascular supply [16–18]. Among them, the laterally closed tunnel (LCT) technique has demonstrated predictable root coverage outcomes and favorable esthetic results, owing to the preservation of tissue integrity, enhanced vascularization, and improved healing potential [19–22]. The LCT has been associated with reduced postoperative pain and higher patient satisfaction compared with conventional flap designs [23, 24]. Nevertheless, the technique requires advanced surgical skills to ensure proper tissue handling and graft stabilization, which may influence clinical predictability [18, 25].

When combined with the CTG, LCT has achieved high success rates, including complete root coverage and stable soft tissue volume augmentation [26]. Despite these advantages, the use of CTG remains limited by donor-site morbidity and increased operative time. Consequently, collagen matrices (CMs) have emerged as a viable alternative, offering reduced patient morbidity while supporting soft tissue regeneration through cellular adhesion and vascular integration [27–30].

Although both CTG and CM have been successfully used with LCT for root coverage, comparative evidence remains scarce, and long-term data regarding the stability of CM outcomes are limited.

Therefore, the present randomized clinical trial aimed to compare the clinical effectiveness and patient-reported outcomes of the LCT technique in combination with either CTG or CM for the management of RT1 localized gingival recession. The null hypothesis was that there would be no significant difference between the two approaches in terms of clinical and patient-centered outcomes.

## Materials and methods

### Study design

This randomized controlled trial (RCT) employed a two-arm parallel group design and included forty patients with RT1 localized defects [4]. All patients were fully informed about the study protocol and procedures, and written informed consent was obtained before enrollment. The trial was approved by the Faculty of Dentistry, Mansoura University, Egypt (Ethical approval number A04060722) and retrospectively registered on ClinicalTrials.gov (ID: NCT06065774). The study was carried out in the Department of Oral Medicine and Periodontology, Faculty of Dentistry, Mansoura University, Egypt, in accordance with CONSORT guidelines [31], and Patient recruitment started in July 2022, and the study was completed in September 2024.

### Population

Patients included in the study were systemically healthy adults aged between 18 and 45 years, presenting with Cairo RT1 gingival recession defect exceeding 4 mm in depth. In cases where minor recessions (< 2 mm) were present on the adjacent teeth, only the primary isolated defect was considered for treatment and analysis. All participants were required to demonstrate a full-mouth plaque score < 15% and to maintain good oral hygiene throughout the study period. Exclusion criteria included the presence of interproximal bone loss at the selected site, pregnancy or lactation, smoking more than 10 cigarettes per day, and any systemic conditions that could interfere with periodontal healing. Sites exhibiting local factors such as cervical restorations, abrasion, non-carious cervical lesions (NCCL), caries, or acute gingival infections were also excluded. Participants with poor oral hygiene, defined as full-mouth plaque or bleeding scores ≥ 15% were not eligible for inclusion [32].

### Randomization, allocation, and blinding

A computerized prepared list of participants was used for the randomization process. Investigator RZ prepared and numbered the sealed opaque envelopes from 1 to 40. The enrollment process was done by randomly withdrawing an envelope from a container just before surgery by another investigator (MS) for unbiased group allocation. A third and fourth investigator (JY and WS), who were uninvolved in the patient’s allocation, assessed all the primary and secondary outcomes. The same experienced surgeon (AE) who was uninvolved in preparation, allocation, or assessment performed all the surgical procedures of the LCT technique with either CTG or CM.

### Sample size calculation

The mean root coverage percentage (MRC%) was used to measure the sample size as reported in previous studies that used the LCT technique in combination with CTG [14, 33]. The MRC% was 96.4% ± 9.4% in patients treated with LCT and CTG (*p* < 0.05) [33]. Using G*Power software (version 3.1.9.4), the sample size was calculated, achieving an effect size of 1.42, with a two-tailed test, α error level of 0.05, and statistical power of 87%. To achieve at least 80% of power to detect a 20% difference, a minimum of 18 subjects per group was required. To compensate for the possible patient dropouts during the study, the final sample size was increased to 40 subjects, allocating 20 subjects for each group.

### Intervention

Professional debridement and cleaning were performed for all participants to prepare the recession site before surgical intervention. The patients were instructed to keep a high level of oral hygiene throughout the study. The patients were unbiasedly allocated between the control (LCT + CTG) and test group (LCT + CM) to ensure randomization.

**Fig. 1:**
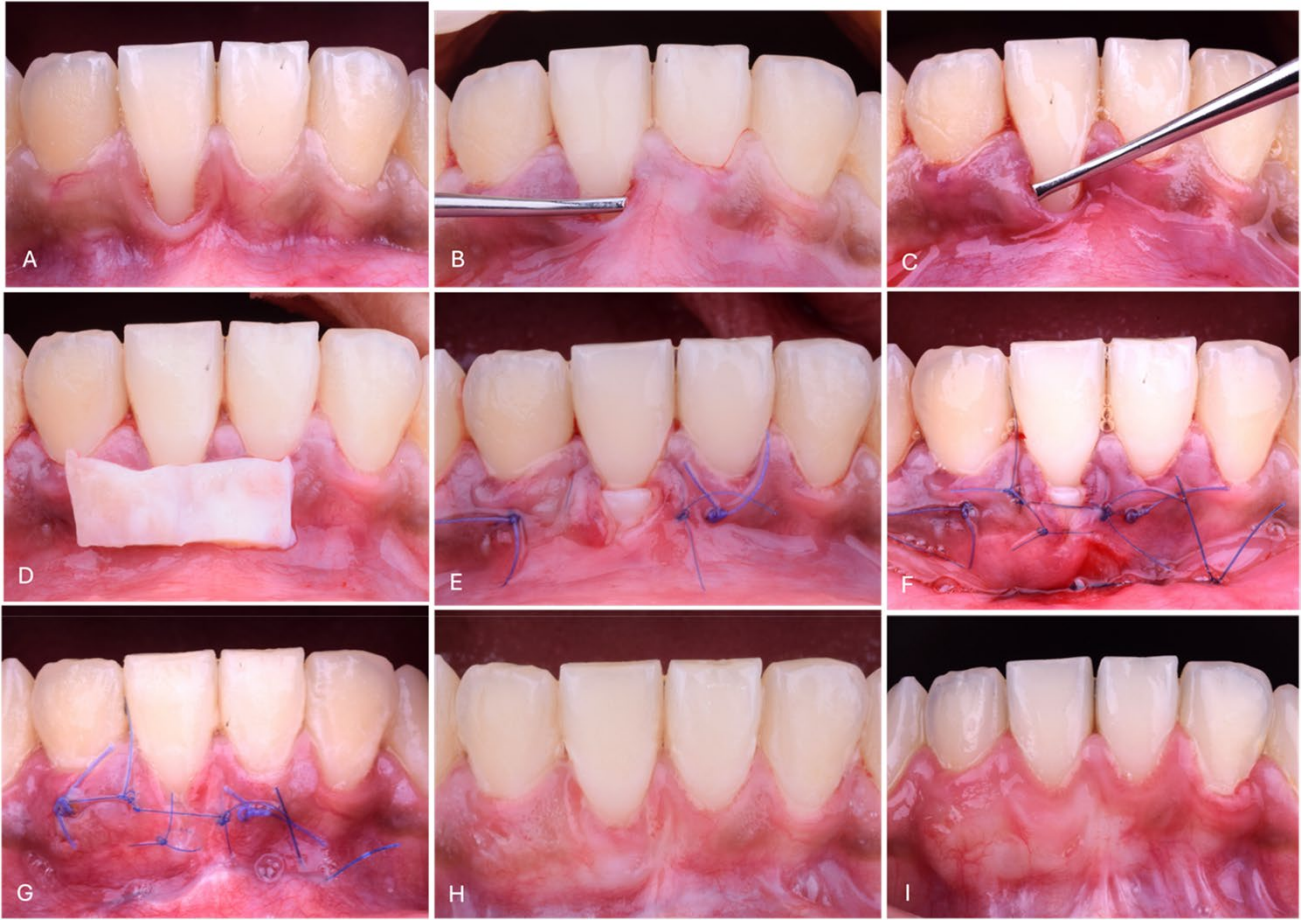
Laterally closed tunnel (LCT) technique with connective tissue graft (CTG) for treatment of localized RT1 gingival recession. **A** Baseline clinical view preoperative; **B**-**C**) Tunnel preparation and tension-free mobilization; **D**) Harvested de-epithelialized CTG; **E**) CTG positioning and fixation within the tunnel; **F**) Final wound closure using modified sling suture and interrupted sutures; **G**) Early post-operative healing; **H**) Clinical outcome at 3 months; **I**) Clinical outcome at 6 months

**Fig. 2:**
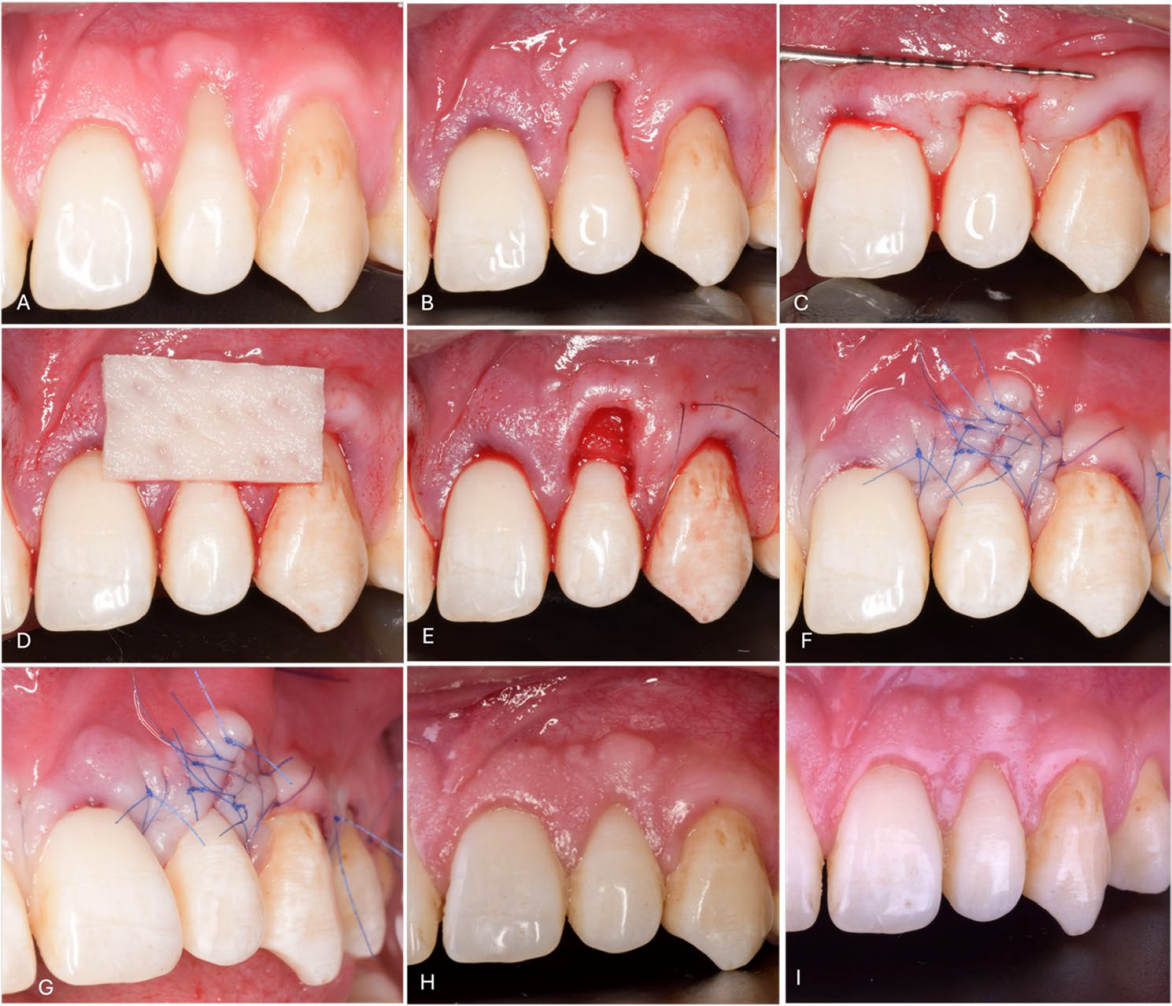
Laterally closed tunnel (LCT) technique with collagen matrix (CM) for treatment of localized RT1 gingival recession. **A** Baseline clinical view preoperative; **B**-**C**) Tunnel preparation and tension-free mobilization; **D**) Xenogeneic collagen matrix (Mucoderm); **E**) CM positioning and fixation within the tunnel; **F**-**G**) Final wound closure using modified sling suture and interrupted sutures; **H**) Clinical outcome at 3 months; **I**) Clinical outcome at 6 months

Local anesthesia (Articaine 4% 1:100,000 Septanest, Septodont, Saint-Maur-des-Fossés, France) was administered. A microsurgical blade (Swan Morton, SM 69, Sheffield, England) was used to make a para-marginal incision through the gingival sulcus to remove the sulcular epithelium. Tunneling instruments (Helmut Zepf, 46.040.30, Seitingen-Oberflacht, Germany) with specific angles were used to make a full-thickness or a partial-thickness tunnel preparation beneath the interdental papilla. The tunnel was extended beyond the MGJ till a tension-free mesial and distal tunnel was created. The margins of the tunnel were approximated over the root surface. The graft material can be easily positioned and fixed to treat the GR defects through the tension-free tunnel (Figs. 1 and 2) [17].

For the CTG group, a 1–1.5 mm thickness de-epithelialized CTG was harvested from the lateral hard palate. Positioning suture (6 − 0 Polypropylene Monofilament, Assut Medical Sarl, Switzerland) was used to make the CTG in the proper position (Fig. 1). Additionally, modified sling sutures (6 − 0 Polypropylene Monofilament, Assut Medical Sarl, Switzerland) were used to fix the graft to the inner part of the tunnel, nearly at the CEJ level. The gingival margins of the tunnel were laterally approximated to cover the CTG partially or completely without tension using simple interrupted sutures (6 − 0 Polypropylene Monofilament, Assut Medical Sarl, Switzerland) (Fig. 1).

In the CM group, the CM (Mucoderm, Botiss, Zossen, Germany) was shaped to match the dimensions of the GR defect with an approximate size of 15 × 10 mm. The CM had to be large enough to cover the adjacent teeth of the recession defect to ensure proper graft vascularization (Fig. 2). Then, the CM was placed in sterile saline solution for 20 min for hydration to facilitate graft insertion into the tunnel without soft tissue trauma. Positioning and fixation sutures were used to place and fix the graft into the proper position underneath the tunnel with the same techniques and suture materials used with the CTG (Fig. 2).

### Postsurgical protocol

Following the surgery, the patients were instructed to avoid trauma to the surgical site by refraining from tooth brushing in the treated site for 2 weeks; instead, they used 0.1% chlorhexidine mouth rinse (OROVEX-H, MACRO GROUP PHARMACEUTICALS, Egypt) twice daily. A professional plaque was provided every 3 days for 2 weeks postoperatively. Analgesics (Brufen 600, ibuprofen, Hakeem Pharmaceuticals, Egypt) and antibiotics (amoxicillin/clavulanic acid, Augmentin 1000 mg, GlaxoSmithKline, UK) were prescribed twice daily for 5 days. In the CTG group, after 1 week, the palatal sutures were removed, whereas the sutures at the treated recession site were removed between 14 and 21 days. Follow-up appointments were scheduled to assess the clinical parameters and healing potentials (1, 3, and 6 months postoperatively). Supportive periodontal therapy and regular oral hygiene instructions were provided to all participants.

### Clinical assessments

All the clinical assessments were performed by two independent and blinded investigators (JY and WS). Clinical measurements were obtained using a periodontal probe (UNC-15, Medesy, Maniago, Pordenone, Italy) at baseline, 3, and 6 months postoperatively. To validate the examiner’s reliability, both inter- and intra-examiner calibration process was performed. For the inter-examiner calibration, both investigators performed the measurements on the same site at the same appointment, and the level of agreement was assessed. For the intra-examiner calibration, each investigator repeated the measurements on the same site twice at 2 different time points after 48-hour intervals. The inter-class correlation coefficient (ICC) demonstrated a high level of reproducibility for all parameters (ICC > 0.90 for intra-examiner calibration, and ICC > 0.85 for inter-examiner calibration).

#### Primary outcome variables

The primary outcome variables included measurements of recession depth (RD), recession width (RW), mean root coverage percentage (MRC%), and complete root coverage percentage (CRC%) to assess the degree of root coverage after 6 months postoperatively.

RD was measured from CEJ to the free gingival margin (FGM), RW was measured horizontally from mesial to distal CEJ point angles, and MRC% was calculated as follows: pre-operative recession depth- post-operative recession depth/pre-operative recession depth×100. While the CRC% % = Total number of treated sites/Number of sites with 100% root coverage​×100. To assess the percentage of sites with complete root coverage [34].

#### Secondary outcome variables

Secondary outcomes included gingival thickness (GT), keratinized tissue width (KTW), clinical attachment level (CAL), attached gingiva width (AGW), root coverage esthetic score (RES), and patient-reported outcome measures (PROMs). These parameters were used to evaluate the short-term periodontal and patient perception following treatment.

The GT was measured using the transgingival probing technique under local anesthesia. A sterile endodontic K-file (ISO 15) (MANI, Inc., Utsunomiya, Tochigi, Japan) with a silicon stopper was inserted perpendicularly at the mid-facial aspect of the treated site, 2 mm apical to the gingival margin, until hard tissue resistance was felt. The penetration depth was recorded to the nearest 0.1 mm with a digital caliper, and the mean of two readings was used for analysis [35].

KTW was measured as the distance from the FGM to the MGJ, while the periodontal probing depth (PPD) was assessed from the FGM to the base of the gingival sulcus using the periodontal probe (UNC-15, Medesy, Maniago, Pordenone, Italy). The CAL was measured from the CEJ to the base of the gingival sulcus, and AGW was calculated by subtracting the PPD from the KTW.

The root coverage esthetic score (RES) was determined according to Cairo et al., evaluating the gingival margin level, contour, soft tissue texture, color, and MGJ alignment with a total score ranging from 0 to 10 (higher score indicating esthetics) [36].

The patient-reported outcome measures (PROMs) included postoperative pain and patient satisfaction. Discomfort was recorded using visual analog scale (VAS, 0–10) at 1 day, 1 week, and 2 weeks post-surgery (0 = no pain; 10 = worst pain). Satisfaction was assessed through a 0–10 scale (0 = not satisfied; 10 = completely satisfied) and a yes/no questionnaire [36–39].

### Statistical analysis

Statistical analysis was conducted using IBM SPSS software version 20.0 (Armonk, NY: IBM Corp., Armonk, USA). A significant level of 5% (*p* < 0.05) was used for statistical relevance. Frequencies and percentages were used to present categorical variables. For continuous variables, the Shapiro-Wilk test was used to assess normality. Descriptive statistics include range (minimum and maximum), mean, standard deviation, median, and interquartile range (IQR) were used for quantitative data.

The following statistical tests were used: the Chi-square test was used to analyze categorical variables between groups, such as site achieved complete root coverage; the student’s t-test was used to compare normally distributed data between two groups such as RD, RW, KTW, and CAL; while for non-normally distributed data, the Mann-Whitney U test was used to assess the difference between groups. The Friedman test was used for comparing non-parametric data across more than two time points, and post hoc pairwise comparisons were done using Dunn’s test [40]. For MRC%, both mean ± SD and the proportions of sites achieving complete root coverage were reported. Both inter- and intra-examiner calibration were assessed using the interclass correlation coefficient (ICC) with value > 0.85 considered excellent.

## Results

### Distribution of defects

**Fig. 3:**
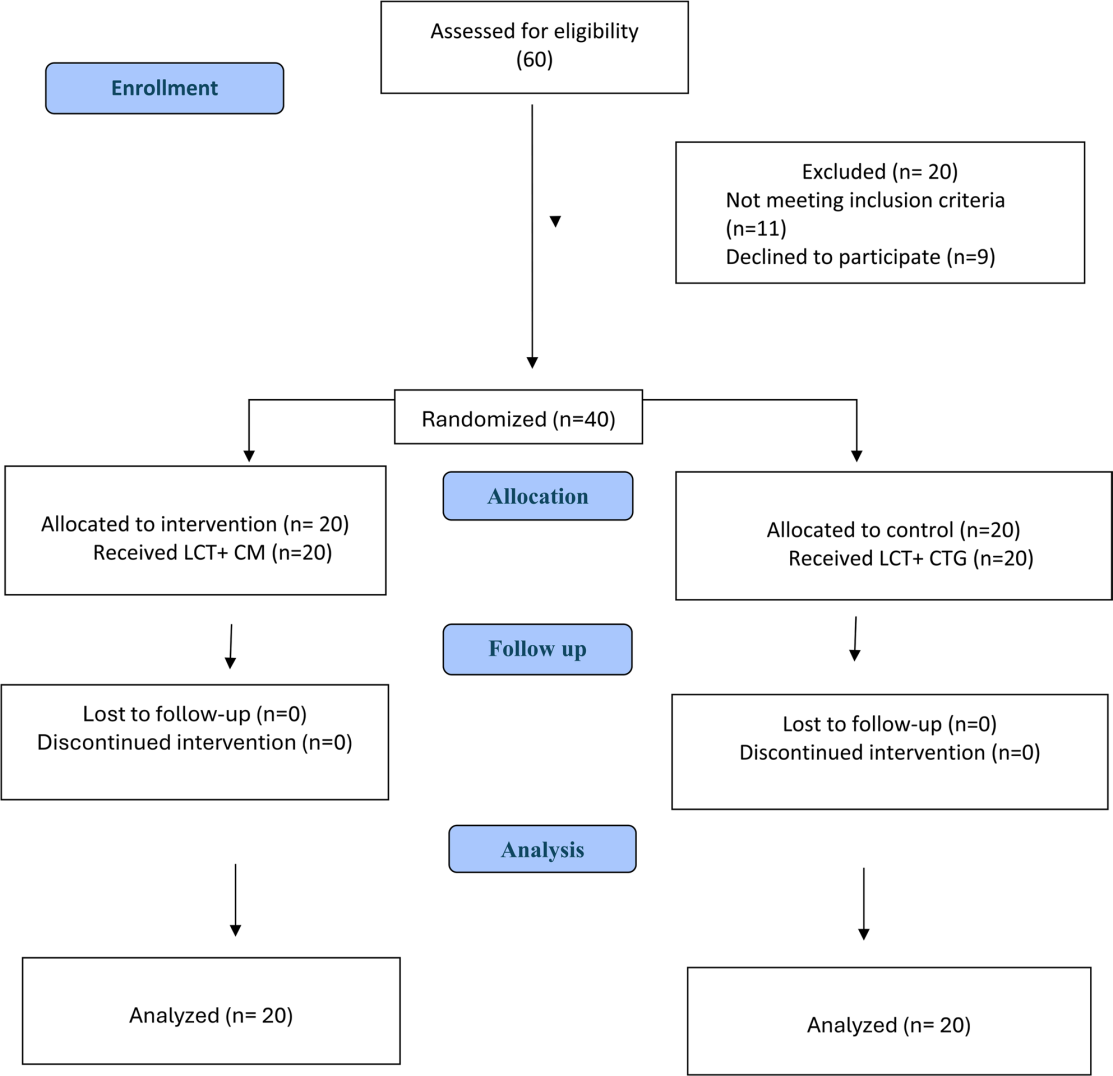
CONSORT 2025 Flow Diagram. Consolidated Standards of Reporting Trials (CONSORT) flow chart for trial

Based on the predetermined inclusion and exclusion criteria, a total of 60 patients were initially screened for eligibility. Following that, 20 patients were excluded: 11 did not fulfill the inclusion criteria, and 9 refused to participate after discussing the study protocol. Ultimately, 40 patients were enrolled in the study after they met the eligibility criteria of the study. The details of the enrollment and allocation process are presented in the CONSORT flow diagram (Fig. 3).

All 40 patients enrolled in the study (19 women and 21 men, mean age 30.05 ± 6.89 years) were successfully randomized into either the control (LCT + CTG) or the test group (LCT + CM). Each patient completed the full duration of the study (July 2022 to September 2024), from the baseline to the final follow-up 6-month period. The baseline recession depth was 5.10 ± 1.74 mm in the control group, and 4.05 ± 1.10 mm in the test group (*P* = 0.052), indicating a non-significant trend toward deeper initial defects in the control group. In 3 of the 40 recessions, the graft was intentionally left exposed through the tunnel, and open healing was observed in the CTG group, with no instance of graft necrosis in the treated sites. Baseline demographic and clinical characteristics are reported in Table 1.

**Table 1:** Comparison between the two groups studied according to demographic data

Demographic data	LCT+CTG(*n* = 20)	LCT+ MUCODERM (*n* = 20)	Test of Significance. (*p*-value)
	No. (%)	No. (%)	
Sex			
Male	11 (55.0%)	10 (50.0%)	0.752
Female	9 (45.0%)	10 (50.0%)	
Age (years)			
Mean ± SD.	30.05 ± 6.75	32.05 ± 6.89	0.359
Median (Min. – Max.)	28.50 (20.0 – 41.0)	33.0 (21.0 – 45.0)	
Location of gingival recession			0.340
Maxillary recessions			
Central incisor	2 (10.0%)	4 (20.0%)	
Lateral incisor	3 (15.0%)	3 (15.0%)	
Canine	2 (10.0%)	3 (15.0%)	
Premolar	2 (10.0%)	3 (15.0%)	
molar	0 (0.0%)	0 (0.0%)	
Mandibular recessions			
Central incisor	4 (20.0%)	3 (15.0%)	
Lateral incisor	3 (15.0%)	2 (10.0%)	
Canine	2 (10.0%)	2 (10.0%)	
Premolar	2(10.0%)	0 (0.0%)	
molar	0 (0.0%)	0 (0.0%)	

*SD* Standard deviation, *t* Student t-test, *c*^*2*^ Chi square test for test of significance, *p* p value for comparing between the two studied groups, *LCT* later closed tunnel, *CTG* Connective tissue graft

### Clinical parameters

#### Primary outcome variables analysis

The primary outcomes focused on the short-term efficacy of the LCT technique for root coverage. Significant improvements in the RD, RW, MRC%, and CRC% were observed from baseline to both 3- and 6-month follow-up intervals in both groups (*p*-value < 0.05; Table 2).

**Table 2:** Comparison between the two groups studied according to primary outcomes in each period

Primary outcomes	LCT + CTG (*n* = 20)			LCT + MUCODERM (*n* = 20)			(Inter group analysis)			Baseline to After 3 M		Baseline to After 6 M	
	Baseline	After 3 M	After 6 M	Baseline	After 3 M	After 6 M	*p*_1_	*p*_2_	*p*_3_	LCT + CTG	LCT + MUCODERM	LCT + CTG	LCT + MUCODERM
Recession depth (RD)	5.10 ± 1.74	0.55 ± 0.51	0.35 ± 0.49	4.05 ± 1.10	1.05 ± 0.51	0.60 ± 0.50	0.052	0.018^*^	0.369	< 0.001^*^	< 0.001^*^	< 0.001^*^	< 0.001^*^
Recession width (RW)	2.40 ± 0.50	0.70 ± 0.73	0.55 ± 0.83	2.35 ± 0.59	1.0 ± 0.46	0.65 ± 0.75	0.883	0.149	0.583	< 0.001^*^	< 0.001^*^	< 0.001^*^	< 0.001^*^
Mean root coverage% (MRC %)	**0.0 ± 0.0**	**90.15 ± 9.70**	**93.98 ± 8.58**	**0.0 ± 0.0**	**74.64 ± 10.92**	**87.24 ± 13.70**	**1.000**	< 0.001^*^	**0.114**	< 0.001^*^	< 0.001^*^	< 0.001^*^	< 0.001^*^
Complete root coverage (CRC%)	0.0 ± 0.0	85%	90%	0.0 ± 0.0	80%	90%	1.000	0.705	1.000	< 0.001^*^	< 0.001^*^	< 0.001^*^	< 0.001^*^

Data was expressed using Mean ± SD

*SD* Standard deviation, *LCT* later closed tunnel, *CTG *Connective tissue graft, *RD *recession depth, *RW* recession width, *MRC%* mean root coverage percentage, *CRC% *Complete root coverage percentage

p_1_: *p* value for Mann Whitney test for comparing between two groups at baseline

p_2_: *p* value for Mann Whitney test for comparing between two groups After 3 months

p_3_: *p* value for Mann Whitney test for comparing between two groups After 6 months

*: Statistically significant at *p*≤ 0.05

At 6 months, nearly complete root coverage was achieved in both groups, with the MRC% exceeding 85% and more than 90% of sites exhibiting complete root coverage. Intragroup analysis confirmed significant gain across all time points (*P* < 0.001). Although the intergroup comparison revealed a significant difference in RD and MRC% at 3 months, favoring the control group, no statistically significant differences were detected between groups for any primary outcomes at 6 months (Table 2).

#### Secondary outcome variables analysis

Secondary outcomes aimed to assess the LCT technique’s predictability in achieving the short-term periodontal stability of the treated sites, as well as the patient’s satisfaction and perception of the procedure. Significant improvements were observed in GT, KTW, CAL, AGW, RES, and PROMs in both groups from baseline to 3- and 6-month follow-up periods (*p*-value < 0.05; Tables 3 and 4).

**Table 3:** Comparison between the two studied groups according to Secondary outcomes in each period

Secondary outcomes	LCT+CTG (*n* = 20)			LCT+ MUCODERM (*n* = 20)			(Intergroup analysis)			Baseline to After 3M		Baseline to After 6M	
	Baseline	After 3M	After 6M	Baseline	After 3M	After 6M	p_1_	p_2_	p_3_	LCT+CTG	LCT+ MUCODERM	LCT+CTG	LCT+ MUCODERM
Gingival thickness (GT)	0.70 ± 0.44	2.60 ± 0.60	2.85 ± 0.49	0.70 ± 0.44	2.50 ± 0.51	2.75 ± 0.44	1.000	0.698	0.640	<0.001^*^	<0.001^*^	<0.001^*^	<0.001^*^
Keratinized tissue width (KTW)	0.95 ± 0.81	3.20 ± 0.70	3.40 ± 0.60	0.80 ± 0.59	2.50 ± 0.51	2.80 ± 0.41	0.718	0.004^*^	0.009^*^	<0.001^*^	<0.001^*^	<0.001^*^	<0.001^*^
Clinical attachment level (CAL)	6.95 ± 2.31	1.70 ± 0.66	1.55 ± 0.60	5.80 ± 1.54	2.15 ± 0.59	1.75 ± 0.64	0.108	0.052	0.369	<0.001^*^	<0.001^*^	<0.001^*^	<0.001^*^
Attached gingiva width (AGW)	-0.90 ± 1.13	2.05 ± 0.76	2.20 ± 0.77	-0.95 ± 0.93	1.40 ± 0.50	1.60 ± 0.50	0.862	0.009^*^	0.020^*^	<0.001^*^	<0.001^*^	<0.001^*^	<0.001^*^
Root coverage esthetic score (RES)	2.20 ± 0.77	8.35 ± 1.53	9.10 ± 1.41	1.85 ± 0.67	7.30 ± 0.92	8.50 ± 1.54	0.165	0.060	0.289	<0.001^*^	<0.001^*^	<0.001^*^	<0.001^*^
	0.40 ± 0.50	0.0 ± 0.0	0.0 ± 0.0	0.20 ± 0.41	0.0 ± 0.0	0.0 ± 0.0	0.289	1.000	1.000	0.058	0.343	0.058	0.343
Plaque index (PI)	0.0 ± 0.0	0.15 ± 0.37	0.0 ± 0.0	0.0 ± 0.0	0.25 ± 0.44	0.0 ± 0.0	1.000	0.602	1.000	0.477	0.236	0.477	1.000
Pink esthetic score(PES)	9.60 ± 1.73	13.45 ± 0.51	13.65 ± 0.49	9.0 ± 1.12	13.50 ± 0.51	13.50 ± 0.51	0.529	0.060	0.429	<0.001^*^	<0.001^*^	<0.001^*^	<0.001^*^
Periodontal Probing depth (PPD)	1.85 ± 0.75	1.15 ± 0.37	1.20 ± 0.41	1.75 ± 0.64	1.10 ± 0.31	1.20 ± 0.41	0.738	0.799	1.000	0.002^*^	0.003^*^	0.003^*^	0.011^*^

Data was expressed using Mean ± SD

*SD* Standard deviation, *LCT* later closed tunnel, *CTG* Connective tissue graft, *GT* gingival thickness, *KTW* keratinized tissue width, *CAL* clinical attachment level, *AGW* attached gingiva width, *RES* root coverage esthetic score

p_1_: *p* value for Mann Whitney test for comparing between two groups at baseline

p_2_: *p *value for Mann Whitney test for comparing between two groups After 3 months

p_3_: *p *value for Mann Whitney test for comparing between two groups After 6 months

*: Statistically significant at *p*≤ 0.05

**Table 4:** Comparison between the two studied groups according to Patient-reported outcome measures (PROMs)

Patient-reported outcome measures PROMs	0.0 ± 0.0	8.70 ± 0.98	9.25 ± 0.64	0.0 ± 0.0	7.70 ± 0.66	8.40 ± 0.75	1.000	0.002^*^	0.002^*^	< 0.001^*^	< 0.001^*^	< 0.001^*^	< 0.001^*^

Data was expressed using Mean ± SD

*SD* Standard deviation, *PROMs *Patient-reported outcome measures

p_1_: *p *value for Mann Whitney test for comparing between two groups at baseline

p_2_: *p *value for Mann Whitney test for comparing between two groups After 3 months

p_3_: *p *value for Mann Whitney test for comparing between two groups After 6 months

*: Statistically significant at *p*≤ 0.05

The GT and KTW increased substantially in both groups, with slightly higher mean values in the control group at 6 months. Additionally, the mean CAL and AGW demonstrated significant gain within each group, with no intergroup difference for CAL. (*P* < 0.001; Table 3)

Esthetic outcomes assessed by the RES were high in both groups, including satisfactory contour, color, and tissue integration. At 6-month follow-up, the PROMs showed high satisfaction scores and minimal discomfort. (*P* < 0.001; Tables 4 and 5)

**Table 5:** Comparison of the visual analogue scale (VAS) in each group between the three study periods

	visual analogue scale (VAS)			Fr	*p*
	Baseline	after 1 week	After 2 weeks		
LCT + CTG (*n* = 20)					
Mean ± SD.	4.50 ± 0.51^a^	1.20 ± 0.41^b^	0.0 ± 0.0^c^	40.000^*^	< 0.001^*^
LCT + MUCODERM (*n* = 20)					
Mean ± SD.	4.40 ± 0.50^a^	1.20 ± 0.41^b^	0.0 ± 0.0^c^	40.000^*^	< 0.001^*^

Different superscript letters (a, b, c) within the same row indicate significant differencesbetween time points (*p* < 0.05). Identical letters indicate no significant difference

*SD *Standard deviation, *VAS *Visual analogue scale

*Fr* Friedman test, Sig. bet. Periods was done using Post Hoc Test (Dunn’s)

p: *p *value for comparing between the three studied periods in each group

*: Statistically significant at *p*≤ 0.05

Although the CTG harvesting could potentially cause greater morbidity, the control group did not report higher pain levels compared to the test group; the VAS pain scores at two weeks were comparable between groups (*p* > 0.05; Table 5).

Intragroup analysis of the secondary outcome variables (GT, KTW, CAL, AGW, RES, and PROMs) in both treatment groups revealed a statistically significant improvement from the baseline to the 6-month follow-up period (*P* < 0.001). The intergroup comparison demonstrated a statistically significant difference in KTW, AGW, and PROMs at the 3- and 6-month follow-up periods. However, the remaining variables did not show significant differences in both follow-up periods (Tables 3 and 4).

## Discussion

This randomized controlled clinical trial evaluated the short-term outcomes of the laterally closed tunnel (LCT) technique combined with either CTG or CM for the treatment of localized RT1 gingival recession defects. Both groups showed significant improvements in root coverage and periodontal stability, with high patient satisfaction and comparable esthetic outcomes after six months. These findings reinforce the effectiveness of the LCT approach in achieving predictable root coverage outcomes.

The present study is among the first RCTs to compare the LCT + CTG and LCT + CM for isolated recessions, while also including patient-reported outcome measures. The results confirmed that the LCT technique provides reliable clinical and esthetic outcomes irrespective of the graft material used.

Although the CAF remains the gold standard approach for root coverage, it has inherent limitations in deep or multiple recessions and cases with interproximal attachment loss. Recent developments in the tunneling techniques, supported by microsurgical instruments, have improved precision and minimized flap tension and scarring. The LCT technique, in particular, preserves papilla integrity and vascular supply, providing superior esthetic blending and reduced morbidity compared with CAF. Consistent with previous reports, our findings confirm that the LCT achieves outcomes comparable to CAF for Miller class I/II or Cairo RT1 defects [4, 17, 21, 41]. Moreover, compared with traditional tunnel techniques, the lateral releasing component in the LCT increases the horizontal mobility, enabling tension-free tissue advancement in deep recession defects, reducing the risk of marginal dehiscence and postoperative scarring [41, 42].

The combination of CTGs with the tunneling technique provided stable and predictable outcomes for GR treatment. However, it is associated with second surgical site and donor site morbidity [29]. Therefore, the CM has been investigated as a scaffold for soft tissue regeneration to treat GR [43]. Our findings agreed with Cieślik et al., who evaluated the short-term effectiveness of the CM with the tunnel technique compared to CTG for multiple recession treatment [44]. Furthermore, our findings align with the long-term results of Molnar et al. for the assessment of CM using the tunnelling technique for RT1 defect treatment [45]. Although debates persist regarding the long-term equivalency of soft tissue substitutes, systematic reviews indicate that CM can achieve outcomes approaching those of CTG while improving patient comfort [2, 41, 46, 47].

The comparable performance between CTG and CM in the present trial can be explained by the favorable biological environment provided by the LCT. The intact tunnel maintains vascular supply, enabling rapid graft revascularization. CTG benefits from intrinsic vascularity and cellularity, while CM acts as a collagenous scaffold promoting cell adhesion and angiogenesis. The combination of a tension-free tunnel and a biologically active graft likely contributed to the high mean root coverage values observed (MRC% and CRC%). The phenomenon of creeping attachment over time may further explain the progressive coronal advancement of the gingival margin seen at six months [48, 49].

Partial graft exposure was noted in three cases within the CTG group; however, all the grafts healed uneventfully without evidence of necrosis. This outcome highlights the strength of the LCT approach, where the tunnel maintains a well-vascularized envelope that preserves the healing in the presence of limited graft exposure. The success of such cases depends on the proportion of the graft that remains covered underneath the tunnel. When the covered portion exceeds the exposed part, adequate revascularization is achievable. Moreover, the biological maturity of CTG allows for rapid vascular integration and prevents volume loss when partial exposure occurs. These findings are consistent with reports supporting that the tunneling technique preserves the blood supply, thereby reducing the risk of graft necrosis compared to other flap designs [42, 50].

A slight baseline imbalance in RD values must be acknowledged, as deeper defects may require longer healing time and may potentially influence short-term root coverage values. However, the treatment effect remained consistent, suggesting that the observed clinical improvements were attributable to the LCT technique and graft material rather than differences in the initial defect severity. Previous studies have similarly shown that creeping attachment and progressive soft tissue maturation can offset initial discrepancies over time [49].

Significant gains in GT and KTW observed in both groups support the regenerative potential of the LCT approach. The improved soft tissue dimensions are attributed to the preserved periosteal blood supply and minimal flap elevation, reducing the bone resorption risk. Our findings agree with Zuhr et al., who highlighted the importance of flap design in tunnelling procedures, particularly in improving the vascular supply to the underlying CTG [51, 52]. The enhanced performance of CTG in the clinical parameters may be attributed to its autogenous origin, which genetically possesses a well-organized mature connective tissue architecture offering a suitable environment for soft tissue integration and long-term stability [30]. The CM provided comparable esthetic outcomes and high patient satisfaction, supporting its suitability for patients seeking a less invasive alternative [53].

The PROMs showed minimal post-operative pain and high satisfaction in both groups. Notably, discomfort in the CTG group was not greater despite graft harvesting, likely due to the superficial harvesting technique used. These results correspond with Zuccelli et al. and others’ reports demonstrating that careful donor site management can minimize morbidity and optimize patient experience [29, 54]. 

The present findings highlight the LCT technique as a reliable, minimally invasive option for the treatment of isolated gingival recession defects. Both CTG and CM demonstrated predictable root coverage, esthetic harmony, and patient satisfaction. The CM may serve as a suitable alternative to CTG, particularly in cases where reduced morbidity or shorter surgical time is desired.

This study has some limitations. The sample size was relatively small, with a short-term (6 months) follow-up and single-center design, which may limit generalizability. No adjustment for multiple comparisons was performed; thus, the possibility of type I error cannot be excluded. In addition, potential confounding factors such as gingival phenotype, operator variability, and technique sensitivity were not assessed. Future multicenter RCTs with larger samples and extended follow-up are needed to confirm the long-term stability of the LCT outcomes and to further elucidate the comparative advantages of CTG and CM.

## Conclusions

Within the limitations of this RCT, the LCT technique demonstrated a predictable and stable outcome for the treatment of localized RT1 gingival recession. Both CTG and CM achieved comparable root coverage and esthetic improvement. CTG offered faster tissue maturation, whereas CM provided a less invasive alternative without donor site morbidity. Further long-term studies with larger sample sizes are recommended to validate these findings.

## Supplementary Information

Supplementary Material 1.

## Data Availability

The datasets generated and analyzed during the current study are available from the corresponding authors upon request.

## References

[1] Kassab MM, Cohen REJ. The etiology and prevalence of gingival recession. TjotAda The etiology and prevalence of gingival recession. 2003;134(2):220–5. 10.14219/jada.archive.2003.0137 12636127

[2] Imber J-C, Kasaj AJIdj. Treatment of gingival recession: when and how? Int Dent J. 2021;71(3):178–87. 34024328 10.1111/idj.12617 PMC9275303

[3] Musskopf ML, Rocha J Md, Rösing CKJ. Perception of smile esthetics varies between patients and dental professionals when recession defects are present. Braz Dent J. 2013;24:385–90. 24173262 10.1590/0103-6440201302223

[4] Cairo F, Nieri M, Cincinelli S, Mervelt J, Pagliaro U. The interproximal clinical attachment level to classify gingival recessions and predict root coverage outcomes: an explorative and reliability study: Interproximal CAL for gingival recessions. J Clin Periodontol. 2011;38(7):661–6. 21507033 10.1111/j.1600-051X.2011.01732.x

[5] Chambrone L, Botelho J, Machado V, Mascarenhas P, Mendes JJ, Avila-Ortiz, GJJop. Does the subepithelial connective tissue graft in conjunction with a coronally advanced flap remain as the gold standard therapy for the treatment of single gingival recession defects? A systematic review and network meta‐analysis. JPER. 2022;93(9):1336–52. 10.1002/JPER.22-0167 35451068

[6] Tavelli L, Barootchi S, Nguyen TV, Tattan M, Ravidà A, Wang HL. Efficacy of tunnel technique in the treatment of localized and multiple gingival recessions: a systematic review and meta‐analysis. JJop Efficacy of tunnel technique in the treatment of localized and multiple gingival recessions: A systematic review and meta-analysis. J Periodontal. 2018;89(9):1075–90. 10.1002/JPER.18-0066 29761502

[7] Abdel-Fatah R, Saleh W, El-Sharkawy H. Efficacy of buccal pad fat as a new approach in the treatment of gingival recession: a systematic review. BMC Oral Health. 2024;24(1):768. 38982391 10.1186/s12903-024-04519-9 PMC11232255

[8] Abdel-Fatah R, Saleh W. Efficacy of amniotic membrane with coronally advanced flap in the treatment of gingival recession: an updated systematic review and meta-analysis. JBoh Efficacy of amniotic membrane with coronally advanced flap in the treatment of gingival recession: an updated systematic review and meta-analysis. 2024;24(1):133. 10.1186/s12903-023-03825-y PMC10811943 38273332

[9] Saleh W, Abdelhaleem M, Elmeadawy S. Assessing the effectiveness of advanced platelet rich fibrin in treating gingival recession: a systematic review and meta-analysis. BMC Oral Health. 2024;24(1):1400. 39563291 10.1186/s12903-024-05115-7 PMC11575048

[10] Abdelhaleem M, Saleh W, Elmeadawy S. Treatment of gingival recession with vestibular incision subperiosteal tunnel access and advanced platelet-rich fibrin. BMC Oral Health. 2025;25(1):63. 39806320 10.1186/s12903-024-05398-w PMC11731150

[11] Cairo F, Pagliaro U, Nieri M. Treatment of gingival recession with coronally advanced flap procedures: a systematic review. J Clin Periodontol. 2008;35:136–62. 18724847 10.1111/j.1600-051X.2008.01267.x

[12] Cortellini P, Pini Prato GJP. Coronally advanced flap and combination therapy for root coverage. Clin Strategies Based Sci Evid Clin Experience. 2012;59(1):158–84. 10.1111/j.1600-0757.2011.00434.x 22507065

[13] De Sanctis M, Zucchelli GJJocp. Coronally advanced flap: a modified surgical approach for isolated recession-type defects: three‐year results. J Clin Periodontol. 2007;34(3):262–8. 17309597 10.1111/j.1600-051X.2006.01039.x

[14] Pini Prato G, Pagliaro U, Baldi C, Nieri M, Saletta D, Cairo F, et al. Coronally advanced flap procedure for root coverage. Flap with tension versus flap without tension: a randomized controlled clinical study. J Periodontol. 2000;71(2):188–201. 10711609 10.1902/jop.2000.71.2.188

[15] Nieri M, Rotundo R, Franceschi D, Cairo F, Cortellini P, Pini Prato GJJ. Factors affecting the outcome of the coronally advanced flap procedure: a Bayesian network analysis. J Periodontol. 2009;80(3):405–10. 19254124 10.1902/jop.2009.080146

[16] Elbana A, Saleh W, Youssef J. Comparing connective tissue grafts and collagen matrix in modified coronally advanced tunnel technique for RT1 gingival recession: a randomized controlled clinical trial. BMC Oral Health. 2025;25(1):893. 40462003 10.1186/s12903-025-06259-w PMC12135576

[17] Sculean A, Allen EPJIJP, Dentistry R. The laterally closed tunnel for the treatment of deep isolated mandibular recessions: surgical technique and a report of 24. Cases. 2018;38(4). 10.11607/prd.3680 29889911

[18] Allen EP, Murphy KG, Sculean A. Papilla access tunnel combined with the laterally closed tunnel: a technique for treatment of deep gingival recession in the anterior mandible. JoE, Dentistry R Papilla Access Tunnel Combined With the Laterally Closed Tunnel: A Technique for Treatment of Deep Gingival Recession in the Anterior Mandible. 2025;37(5):1157–64. 10.1111/jerd.13324 39462875

[19] Barootchi S, Tavelli LJIJED. Tunneled coronally advanced flap for the treatment of isolated gingival recessions with deficient papilla. 2022;17(1). 35175005

[20] González-Febles J, Romandini M, Laciar-Oudshoorn F, Noguerol F, Marruganti C, Bujaldón-Daza A, et al. Tunnel vs. coronally advanced flap in combination with a connective tissue graft for the treatment of multiple gingival recessions: a multi-center randomized clinical trial. Clin Oral Invest. 2023;27(7):3627–38. 10.1007/s00784-023-04975-7 PMC10329586 36988824

[21] Lanzrein C, Guldener K, Imber J-C, Katsaros C, Stähli A, Sculean AJQI. Treatment of multiple adjacent recessions with the modified coronally advanced tunnel or laterally closed tunnel in conjunction with cross-linked hyaluronic acid and subepithelial connective tissue graft: a report of 15 cases. 2020;51(9):710–9. 10.3290/j.qi.a44808 32577705

[22] Katti N, Kp R, Barik AK, Das SK, Peri S, Mohanty DJC. <article-title update="added">Enhancing root coverage and esthetic outcomes in isolated gingival recession using orthodontic intervention and lateral closed tunnel technique: an interdisciplinary prospective case series. AiP Enhancing root coverage and esthetic outcomes in isolated gingival recession using orthodontic intervention and lateral closed tunnel technique: An interdisciplinary prospective case series. 2025;15(1):14–24. 10.1002/cap.10285 38526009

[23] Amitha K, Paramashivaiah R, Prabhuji MLV, Subramanya AP, Assiry AA, Peeran SW, et al editors. Clinical assessment of the effects of low-level laser therapy on coronally advanced flap procedure in the management of isolated gingival recession. Photonics: MDPI; 2022.

[24] Skierska I, Górski B, Fus Ł. <article-title update="added">Tunnel technique and subepithelial connective tissue graft, with or without cross‐linked hyaluronic acid, in the treatment of multiple gingival recessions: 12‐month outcomes of a randomized clinical trial. J Periodontol. 2024;95(11):1060–72. 38808976 10.1002/JPER.24-0093

[25] Elbana A, Saleh W, Youssef JJBOH. Comparing connective tissue grafts and collagen matrix in modified coronally advanced tunnel technique for RT1 gingival recession: a randomized controlled. Clin Trial. 2025;25(1):893. 10.1186/s12903-025-06259-w PMC12135576 40462003

[26] Moraschini V, de Almeida DCF, Sartoretto S, Bailly Guimarães H, Chaves Cavalcante I, Diuana Calasans-Maia MJAOS. Clinical efficacy of xenogeneic collagen matrix in the treatment of gingival recession: a systematic review and meta-analysis. 2019;77(6):457–67. 10.1080/00016357.2019.1588372 30896271

[27] Jepsen K, Jepsen S, Zucchelli G, Stefanini M, de Sanctis M, Baldini N, et al. Treatment of gingival recession defects with a coronally advanced flap and a xenogeneic collagen matrix: a multicenter randomized clinical trial. J Clin Periodontol. 2013;40(1):82–9. 23050490 10.1111/jcpe.12019

[28] Puri K, Kumar A, Khatri M, Bansal M, Rehan M, Siddeshappa STJ. 44-year journey of palatal connective tissue graft harvest: a narrative review. J Indian Soc Periodontol. 2019;23(5):395–408. 31543611 10.4103/jisp.jisp_288_18 PMC6737854

[29] Zucchelli G, Mele M, Stefanini M, Mazzotti C, Marzadori M, Montebugnoli L, et al. Patient morbidity and root coverage outcome after subepithelial connective tissue and de-epithelialized grafts: a comparative randomized‐controlled clinical trial. J Clin Periodontol. 2010;37(8):728–38. 20590963 10.1111/j.1600-051X.2010.01550.x

[30] Barootchi S, Tavelli L, Zucchelli G, Giannobile WV, Wang HL. Gingival phenotype modification therapies on natural teeth: a network meta-analysis. J Periodontol. 2020;91(11):1386–99. 32392401 10.1002/JPER.19-0715

[31] Hopewell S, Chan A-W, Collins GS, Hróbjartsson A, Moher D, Schulz KF, et al. CONSORT 2025 statement: updated guideline for reporting randomised trials. Lancet. 2025;405(10489):1633–40. 10.1016/S0140-6736(25)00672-5 40245901

[32] Tankova H, Lazarova Z. A comparative study of full mouth bleeding score (FMBS) and gingival index Loe and Silness (GILS) in assessing gingival status of children aged 10–14 years. J IMAB. 2024;30(1):5387–91.

[33] Rotundo R, Pini Prato GP. Use of a new collagen matrix (mucograft) for the treatment of multiple gingival recessions. JTIjop, dentistry r. 2012;32:413–9. 22577647

[34] Zucchelli G, Testori T, De Sanctis MJ. Clinical and anatomical factors limiting treatment outcomes of gingival recession: a new method to predetermine the line of root coverage. J Periodontol. 2006;77(4):714–21. 16584355 10.1902/jop.2006.050038

[35] Kloukos D, Koukos G, Gkantidis N, Sculean A, Katsaros C, Stavropoulos A. Transgingival probing: a clinical gold standard for assessing gingival thickness. Quintessence Int. 2021;52(5):394–401. 33533238 10.3290/j.qi.b937015

[36] Cairo F, Rotundo R, Miller PD Jr. Pini Prato GPJJop. Root coverage esthetic score: a system to evaluate the esthetic outcome of the treatment of gingival recession through evaluation of clinical cases. 2009;80(4):705 – 10. 10.1902/jop.2009.080565 19335093

[37] Korkmaz B, Balli UJC. Clinical evaluation of the treatment of multiple gingival recessions with connective tissue graft or concentrated growth factor using tunnel technique: a randomized controlled clinical trial. 2021:1–10. 10.1007/s00784-021-03935-3 33830339

[38] Fürhauser R, Florescu D, Benesch T, Haas R, Mailath G, Watzek GJ. Evaluation of soft tissue around single‐tooth implant crowns: the pink esthetic score. Clin Oral Implants Res. 2005;16(6):639–44. 16307569 10.1111/j.1600-0501.2005.01193.x

[39] Weldring T, Smith SMJH. Article commentary: patient-reported outcomes (PROs) and patient-reported outcome measures (PROMs). 2013;6:HSI. S11093. 10.4137/HSI.S11093 PMC4089835 25114561

[40] Quirk TJ. Quirk TJJEfe, problems psAgtsp. One-way analysis of variance (ANOVA). 2012:163 – 79.

[41] Toledano-Osorio M, Muñoz-Soto E, Toledano M, Vallecillo-Rivas M, Vallecillo C, Ramos-García P, et al. Treating gingival recessions using coronally advanced flap or tunnel techniques with autografts or polymeric substitutes: a systematic review and meta-analysis. Polymers. 2022;14(7):1453. 35406326 10.3390/polym14071453 PMC9002830

[42] Stähli A, Dent M, Miron RJ, Deppe H, Dent DM, Cosgarea R et al. The combined laterally closed, coronally advanced tunnel for the treatment of mandibular multiple adjacent gingival recessions: surgical technique and a report of 11 cases. 2021;52:576. 10.3290/j.qi.b1098307 33749221

[43] Zegarra-Caceres L, Orellano-Merluzzi A, Muniz FWMG, de Souza SLS, Faveri M, Meza-Mauricio JJO. Xenogeneic collagen matrix vs. connective tissue graft for the treatment of multiple gingival recession: a systematic review and meta-analysis. 2024;112(2):317–40. 10.1007/s10266-023-00863-4 37898589

[44] Cieślik-Wegemund M, Wierucka‐Młynarczyk B, Tanasiewicz M, Gilowski ŁJJop. Tunnel technique with collagen matrix compared with connective tissue graft for treatment of periodontal recession: a randomized clinical trial. 2016;87(12):1436–43. 10.1902/jop.2016.150676 27424564

[45] Molnár B, Aroca S, Dobos A, Orbán K, Szabó J, Windisch P, et al. Treatment of multiple adjacent RT 1 gingival recessions with the modified coronally advanced tunnel (MCAT) technique and a collagen matrix or palatal connective tissue graft: 9-year results of a split-mouth randomized clinical trial. Clin Oral Invest. 2022;26(12):7135–42. 10.1007/s00784-022-04674-9 PMC9708797 35994126

[46] Chauca-Bajaña L, Pérez-Jardón A, Silva FFVE, Conde-Amboage M, Velásquez-Ron B, Padín-Iruegas E, et al. Root coverage techniques: coronally advancement flap vs. tunnel technique: a systematic review and meta-analysis. Dent J (Basel). 2024;12(11):341. 39590391 10.3390/dj12110341 PMC11592634

[47] Mayta-Tovalino F, Barboza JJ, Pasupuleti V, Hernandez AV. Efficacy of tunnel technique (TUN) versus coronally advanced flap (CAF) in the management of multiple gingival recession defects: a meta-analysis. JIJoD. 2023;2023(1):8671484. 10.1155/2023/8671484 PMC10101741 37063452

[48] Assimi S, Ismaili Z, Dghoughi S. Successful management of gingival recession with creeping attachment: a case report. Clin Case Rep. 2024;12(5):e8952. 38756619 10.1002/ccr3.8952 PMC11096279

[49] Prathivi HK, Sari RJMKG. Creeping attachment post-gingival recession treatment using a vestibular incision subperiosteal tunneling access technique combined with a connective tissue graft. 2023;56(1):13–6.

[50] Joly JC, Carvalho AM, da Silva RC, Ciotti DL, Cury PR. Root coverage in isolated gingival recessions using autograft versus allograft: a pilot study. J Periodontol. 2007;78(6):1017–22. 17539714 10.1902/jop.2007.060428

[51] Zuhr O, Fickl S, Wachtel H, Bolz W, Hurzeler M. Covering of gingival recessions with a modified microsurgical tunnel technique: case report. JIJoP, Dentistry R. 2007;27(5):457. 17990442

[52] Zuhr O, Bäumer D, Hürzeler MJ. The addition of soft tissue replacement grafts in plastic periodontal and implant surgery: critical elements in design and execution. J Clin Periodontol. 2014;41:S123–42. 24640997 10.1111/jcpe.12185

[53] Pietruska M, Skurska A, Podlewski Ł, Milewski R, Pietruski JJ. Clinical evaluation of Miller class I and II recessions treatment with the use of modified coronally advanced tunnel technique with either collagen matrix or subepithelial connective tissue graft: a randomized clinical study. J Clin Periodontol. 2019;46(1):86–95. 30362599 10.1111/jcpe.13031

[54] Zucchelli G, Tavelli L, McGuire MK, Rasperini G, Feinberg SE, Wang HL, et al. Autogenous soft tissue grafting for periodontal and peri-implant plastic surgical reconstruction. J Periodontol. 2020;91(1):9–16. 31461778 10.1002/JPER.19-0350

